# Health crisis in Venezuela: Status of communicable diseases and implications for the European Union and European Economic Area, May 2019

**DOI:** 10.2807/1560-7917.ES.2019.24.22.1900308

**Published:** 2019-05-30

**Authors:** Laura Espinosa, Grazina Mirinaviciute

**Affiliations:** 1 European Centre for Disease Prevention and Control, Solna, Sweden

**Keywords:** Venezuela, communicable diseases, migrants, measles, malaria, arboviruses

## Abstract

Re-emerging diseases outbreaks are being reported in Venezuela since 2012/13, following ongoing political and economic crisis. Healthcare system collapse has led to an increasing incidence and mortality from communicable diseases. Increasing movement of people between Venezuela and the European Union and European Economic Area (EU/EEA) creates a need for increased awareness of the infectious disease risks and requirements for appropriate investigation and treatment of individuals arriving from Venezuela; overall risk for EU/EEA citizens is low.

Since 2012/13, Venezuela has experienced a rapid deterioration of the public health situation due to an ongoing political and economic crisis [1]. This crisis has led to the collapse of the healthcare system, resulting in the re-emergence of previously controlled or eliminated communicable diseases and to the lack of proper provision of sanitation, goods, services and food security [2,3]. In March–April 2019, recurrent and prolonged electricity blackouts in large parts of the country affected the availability of running water, telecommunication within and outside the country, transportation, adequate food cooling (e.g. fridge/freezers), economic mobility and education [4]. The healthcare sector was affected by the closure of operating theatres and emergency rooms, lack of care for chronic patients requiring medical devices for their survival and lack of medicines and vaccines [4].

Here, we present the latest available information on communicable diseases in Venezuela and the possible implications that it may have in the European Union and European Economic Area (EU/EEA), hereafter referred to as Europe.

## Status of communicable diseases in Venezuela

In December 2016, the latest national report on communicable diseases was published by the Venezuelan Ministry of Health [5], in which an increase in infant and maternal mortality, a resurgence of diphtheria and an increase in the incidence of malaria, Zika, tuberculosis and hepatitis A was reported. The current situation of communicable diseases in Venezuela is available from reports from the World Health Organization (WHO), scientific research and studies/surveys on specific pathogens. Most of these sources acknowledge a noticeable gap between the reported and the estimated incidence or prevalence of some communicable diseases, thus the numbers in Table 1 demonstrate only confirmed cases and should be read with caution of possible underestimation (Table 1).

**Table 1:** Latest available data on confirmed cases of communicable diseases and associated deaths in Venezuela, by disease and time period

Disease	Time period	Confirmed cases	Deaths	Source
**Vaccine preventable diseases**				
**Diphtheria**	W26 2016–W08 2019^a^	1,612	280	PAHO [16]
**Measles**	W26 2017–W52 2017^a^	727	2	PAHO [6]
	2018	5,667	74	PAHO [6]
	W01 2019–W13 2019^b^	140	NA	PAHO [6]
**Mosquito-borne diseases**				
**Chikungunya**	2014	2,303	0	PAHO [17]
	2015	347	0	PAHO [17]
	2016	355	0	PAHO [17]
	2017	39	0	PAHO [17]
**Dengue**	2016	5,833	39	PAHO [18]
	2017	1,588	16	PAHO [18]
	2018	2,440	24	PAHO [18]
	W01 2019–W17 2019^b^	288	8	PAHO [18]
**Malaria**	2016	240,613	NA	WHO [9]
	2017	414,000	NA	WHO [9]
**Zika**	2016	2,380	0	PAHO [19]
	2017	2,413	0	PAHO [19]
**Other vector-borne diseases**				
**Chagas**	Oct 2017–Apr 2018^a^	40	8	Grillet et al. 2019 [7]
**Oral Chagas disease**	May 2016^a^	5	0	Grillet et al. 2019 [7]
	Sep 2017^a^	6	3	Grillet et al. 2019 [7]
	Mar 2018	42	6	Grillet et al. 2019 [7]
**Cutaneous leishmaniasis**	1990–2016^a^	61,576	NA	Grillet et al. 2019 [7]
	2014	Ca 1,750	NA	Grillet et al. 2019 [7]
	2015	Ca 2,000	NA	Grillet et al. 2019 [7]
	2016	Ca 2,000	NA	Grillet et al. 2019 [7]
**HIV and tuberculosis**				
**HIV**	2016	120,000^c^	2,500	UNAIDS [20]
**Tuberculosis**	2017	10,952^c^	800	WHO [21]

HIV: human immunodeficiency virus; NA: not available; PAHO: Pan American Health Organisation; WHO: World Health Organization.

^a ^Time period corresponding to an outbreak.

^b ^Incomplete year.

^c ^These are prevalent confirmed cases.

Only confirmed cases are reported in Table 1. There may be a possible underestimation of cases; therefore, numbers should be interpreted with caution.

### Vaccine preventable diseases

Diphtheria and measles have re-emerged due to vaccine shortages and lack of vaccination programmes, in July 2016 and in 2017, respectively. Between 2016 and 2018, there have been 280 deaths from diphtheria. In 2018, there were over 5,000 confirmed measles cases, with 10% of the cases affecting indigenous populations in Amazonas and Delta Amacuro states [6].

### Mosquito-borne diseases

Arboviruses, e.g. dengue, chikungunya and Zika virus, are an expanding threat in the country [7]. Due to interruptions of water supply and electricity, residents in Venezuela have to store water in receptacles that maintain favourable breeding conditions for *Aedes* mosquitoes. In addition, there is an increased possibility of severe dengue cases due to co-circulation of four dengue virus serotypes (DENV 1–4) [8]. Epidemics of chikungunya in 2014 and Zika virus in 2015–16 reported in Latin America also impacted the country with a high number of cases (Table 1).

Confirmed cases of malaria increased between 2016 and 2017, with 240,613 detected in 2016 and 414,000 malaria cases in 2017 – the highest annual total recorded since 1988 [9]. It has been estimated that there were one million malaria cases in 2018, including non-reported infections and relapses [10]. The surveillance programme against malaria has been gradually dismantled since 2012 [11].

### Other vector-borne diseases

Additional vector-borne diseases have been reported in the country. The seroprevalence of *Trypanosoma cruzi,* which causes Chagas disease, has increased in recent decades following decentralisation of control measures in 1990s and after the Chagas diseases programme was dismantled in 2012 [7]. Between October 2017 and April 2018 there were 40 confirmed cases of Chagas disease (Table 1). Leishmaniasis is widespread in the human and animal population, outbreaks of Mayaro virus have been reported in the past decade, Oropouche virus in the Amazonas state and the occurrence of epizootic strains of Venezuelan equine encephalitis [7].

### HIV and tuberculosis

The latest available information on cases of HIV and tuberculosis from 2016 and 2017, respectively, can be seen in Table 1. The incidence rate of tuberculosis is the highest reported in the country for the past 40 years [12], with more than 10,000 confirmed cases in 2017 (Table 1).

## Asylum seekers and travellers returning from Venezuela

**Figure 1:**
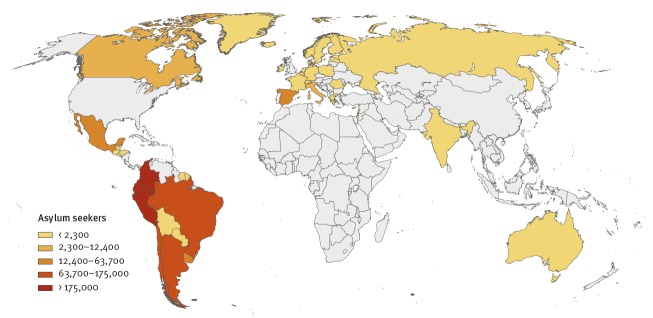
Geographic distribution of asylum seekers from Venezuela by country of asylum, January–June 2018 (n = 1,750,000)

Source: United Nations High Commissioner for Refugees (UNHCR).

An increased emigration of Venezuelan nationals has been reported since 2014 [7]. There were ca 1,750,000 Venezuelans reported to be seeking asylum between January and June 2018, 46% of whom applied for asylum in Peru, Ecuador or Colombia (Figure 1) [13].

In Europe, the number of Venezuelan asylum seekers increased from ca 320 in 2014 to more than 20,000 in 2018, with almost all applications being filed in five countries (Spain, Italy, France, Germany and Belgium) and ca 85% of these in Spain (Figure 1) [14]. According to the latest data from the European Asylum Support Office, there were ca 6,600 Venezuelan nationals applying for asylum in Europe in January–February 2019 [14].

**Figure 2:**
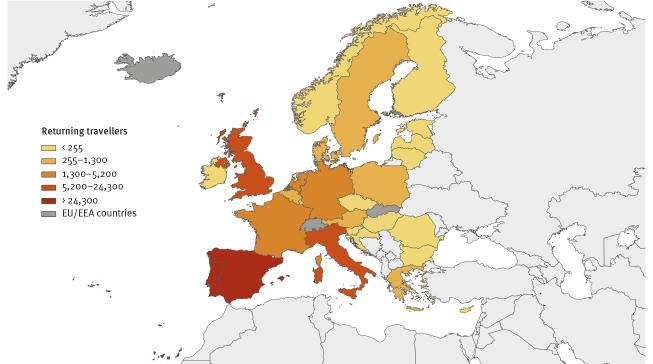
Geographic distribution of travellers returning from Venezuela to the EU/EEA, January–December 2017 (n = 150,000)

In addition, there were ca 150,000 travellers returning from Venezuela to Europe in 2017, according to the International Air Transport Association (IATA) (Figure 2). Of these, 87% were returning to Spain, Portugal or Italy. The available data show the country of departure and destination of the air travel, but not the nationality of the travellers.

EU/EEA: European Union and European Economic Area.

Source: International Air Transport Association (IATA).

## Discussion and conclusions

As a consequence of political and economic crisis, Venezuela faces a re-emergence of communicable diseases that were previously eliminated or controlled. Increasing numbers of people are affected by an increased incidence of infectious diseases (and outbreaks) and mortality, which may be underestimated in the absence of proper surveillance and lack of treatment; thus, the magnitude of the outbreaks may be larger than presented. In addition, the mass departure of trained medical personnel reported in recent years is having an impact on the proper care of patients [7]. People with chronic health conditions, pregnant and nursing women, children under 5 years, indigenous population, people without a fixed residence and those living with disabilities are among the most vulnerable [12].

The migration of people from Venezuela to neighbouring countries and to Europe requires increased awareness that Venezuelans (in particular the aforementioned risk groups) are facing heightened infectious disease risks and that there is a need for appropriate investigation and treatment by healthcare providers in the receiving countries.

The situation in Venezuela and travel to and from the country increases the possibility for European citizens of being exposed to circulating infectious diseases. Previous outbreaks in neighbouring and European countries have been shown to have originated from Venezuela, such as a Chagas disease outbreak in Colombia in 2017–18 [7] and dengue outbreak in Madeira in 2012 [15].

The deterioration in public health systems e.g. vaccination programmes in Venezuela also means that migrants to Europe may be at risk from diseases present in Europe, such as measles. Therefore, Venezuelan migrants residing in Europe should be recommended to seek advice from healthcare providers on vaccination requirements in the country of residence. They should also be advised to seek immediate medical advice and mention that they have recently arrived from Venezuela in case of symptoms suggestive of gastrointestinal, respiratory, cutaneous or any other type of infection. The travellers visiting Venezuela should be appropriately vaccinated, follow good hygiene practices, practice safe sex and use preventive measures against mosquito bites to prevent vaccine-preventable diseases such as measles, food- and water-borne diseases, sexually transmitted infections such as HIV and mosquito-borne diseases such as dengue or malaria, among others. Currently, the overall risk for severe diseases is considered low for European residents and returning travellers from Venezuela if public health advice is followed.
